# Agreement between Vital Signs Measured Using Mat-Type Noncontact Sensors and Those from Conventional Clinical Assessment

**DOI:** 10.3390/healthcare12121193

**Published:** 2024-06-13

**Authors:** Daiki Shimotori, Eri Otaka, Kenji Sato, Munetaka Takasugi, Nobuyoshi Yamakawa, Atsuya Shimizu, Hitoshi Kagaya, Izumi Kondo

**Affiliations:** 1 Laboratory of Practical Technology in Community, Assistive Robot Center, National Center for Geriatrics and Gerontology, Obu 474-8511, Aichi, Japan; d-shimotori@ncgg.go.jp; 2 Department of Rehabilitation, National Center for Geriatrics and Gerontology, Obu 474-8511, Aichi, Japan; k-sato@ncgg.go.jp (K.S.); hkagaya2@ncgg.go.jp (H.K.); ik7710@ncgg.go.jp (I.K.); 3 Techno Horizon Co., Ltd., Nagoya 457-0071, Aichi, Japan; tak@th-grp.jp (M.T.); yamakawa-no@th-grp.jp (N.Y.); 4 Department of Cardiology, National Center for Geriatrics and Gerontology, Obu 474-8511, Aichi, Japan; ashimizu@ncgg.go.jp

**Keywords:** vital signs, non-contact sensor, cardiovascular disease, respiratory rate, pulse rate

## Abstract

Vital signs are crucial for assessing the condition of a patient and detecting early symptom deterioration. Noncontact sensor technology has been developed to take vital measurements with minimal burden. This study evaluated the accuracy of a mat-type noncontact sensor in measuring respiratory and pulse rates in patients with cardiovascular diseases compared to conventional methods. Forty-eight hospitalized patients were included; a mat-type sensor was used to measure their respiratory and pulse rates during bed rest. Differences between mat-type sensors and conventional methods were assessed using the Bland–Altman analysis. The mean difference in respiratory rate was 1.9 breaths/min (limits of agreement (LOA): −4.5 to 8.3 breaths/min), and proportional bias existed with significance (r = 0.63, *p* < 0.05). For pulse rate, the mean difference was −2.0 beats/min (LOA: −23.0 to 19.0 beats/min) when compared to blood pressure devices and 0.01 beats/min (LOA: −11.4 to 11.4 beats/min) when compared to 24-h Holter electrocardiography. The proportional bias was significant for both comparisons (r = 0.49, *p* < 0.05; r = 0.52, *p* < 0.05). These were considered clinically acceptable because there was no tendency to misjudge abnormal values as normal. The mat-type noncontact sensor demonstrated sufficient accuracy to serve as an alternative to conventional assessments, providing long-term monitoring of vital signs in clinical settings.

## 1. Introduction

Vital signs, including respiratory rate, pulse rate, blood pressure, body temperature, oxygen saturation, and level of consciousness, are widely used in clinical settings to quickly assess a patient’s health status [1,2]. These indicators not only help understand a patient’s current health condition but also play a crucial role in assisting diagnosis, evaluating treatment effectiveness, and early detection of symptoms [3,4,5]. Regular monitoring of vital signs is particularly important in patients with cardiovascular diseases. Nevertheless, simply increasing the frequency of vital sign measurements also creates problems of increased workload and the lack of record keeping in clinical settings [6,7]. To address these problems, an increase in personnel or the introduction of long-time recording devices could be considered, but an increase in personnel is not realistic from a cost standpoint. Therefore, 24-h Holter electrocardiogram (ECG) and polysomnography (PSG) are used for onset and common long-term vital sign monitoring tests. These tests allow the measurement and recording of vital signs by directly attaching devices to the body. This eliminates the need for additional personnel to record data and prevents the lack of measured vital sign data. Moreover, these devices enable the continuous monitoring of vital signs, including those during the night. The resting pulse rate at night is not only an important predictor of cardiovascular events [8,9] but also essential for assessing exercise intensity and planning exercise programs [10,11], which is particularly crucial for patients with cardiovascular diseases. However, wearing specialized devices for extended periods can be physically and mentally demanding. Those with cognitive impairments tend to remove these uncomfortable attachments because it is difficult for them to understand the necessity of the attached devices. In addition, maintaining the correct position for accurate measurements is difficult in patients with deteriorated postural stability.

In recent years, noncontact sensor technology has been developed to address these issues and enable continuous monitoring of vital signs [12,13,14]. Noncontact sensors used for vital sign monitoring include cameras [15], radar [16], and thermography [17]. These sensors need to record the movement of the patient’s thorax and facial condition, and the less obstruction between the sensor and the body, the better. However, when the installed sensors are visible to the subject, they may cause mental stress owing to their awareness of being constantly monitored. Mental stress can affect the results of vital recordings [18]. By contrast, mat-type sensors placed underneath the mattress allow vital sign monitoring without the subject’s awareness of being measured. The mat-type sensor measures vital signs by detecting minute physical changes associated with heartbeat and respiration over the bed mattress. In this method, there is no direct contact between the patient and the sensor. Therefore, there is no discomfort from the worn device or mental stress owing to the awareness of being monitored. A recent review compared the performance of existing noncontact sensing technologies for monitoring vital signs and showed that most of the affordable sensors in two main categories, pressure sensors and force sensors, demonstrate a certain level of accuracy [14]. For example, a mat-type device consisting of a thin ferroelectric pressure sensor combined with ballistocardiography (Emfit QS) can reliably measure heart and respiration rates of healthy subjects and patients with sleep disorders in a laboratory setting [19] and those of healthy subjects in a home environment [20]. However, these studies were conducted only on subjects with normal vital signs, and therefore, it is unclear whether mat-type sensors can be applied to cardiovascular patients with abnormal breathing and pulse rates [14]. The verification of mat-type sensors in patients with cardiovascular diseases is crucial, as the continuous monitoring of vital signs enables early detection of deterioration and timely interventions in these high-risk populations. In this regard, a mat-type device using a pressure-detecting method via air vibration [21,22] is considered more suitable for use with these patients in clinical practice in terms of durability and electromagnetic radiation. However, although these new medical devices must be sufficiently validated in the target user population through well-established clinical assessments before being used in clinical practice [14,23], the clinical validation of this air vibration method is yet to be conducted.

Therefore, this study aimed to evaluate the accuracy of respiratory and pulse rates measured using mat-type noncontact sensors with the air vibration method among patients with cardiovascular diseases compared to conventional methods used in clinical practice and to explore the possibility of their clinical application. The use of noncontact vital sign monitoring technology in clinical practice complements conventional measurement methods and enables continuous vital sign monitoring. In doing so, it is expected to play an important role in providing higher-quality medical care without overlooking worsening symptoms.

## 2. Materials and Methods

### 2.1. Study Design and Settings

This study was designed as a validation study to evaluate the accuracy of a mat-type noncontact sensor in measuring respiratory and pulse rates among patients with cardiovascular diseases. We compared the sensor performance to conventional methods used in clinical practice, including visual inspection for respiratory rate, automated blood pressure devices, and 24-h Holter ECG for pulse rate.

The participants in this study were patients with acute or sub-acute cardiovascular diseases (heart failure and stroke) hospitalized at the National Center for Geriatrics and Gerontology, Japan. The inclusion criteria were as follows: patients whose general condition was stable enough to undergo rehabilitation intervention, who could lie in the supine position, who could understand the study procedures, and who provided informed consent to participate. The exclusion criteria were as follows: (1) patients with cognitive impairment (scoring 15 points or less on the Mini-Mental State Examination-Japanese version [MMSE-J] [24]), who were considered to have an insufficient understanding of the study content; (2) patients who could not use the device properly because of difficulty lying in a supine position; (3) patients with unstable symptoms requiring intense treatment; and (4) patients with severe agitation, delirium, or restlessness that may lead to excessive body movements and interfere with the accurate measurement of vital signs.

Through an a priori power analysis for calculating mean difference using G*Power 3.1, we estimated at least a sample size of 34 in total to provide an effect size of 0.5, power of 80%, and a type I error of 0.05 using a two-tailed test.

The study protocol was approved by the Ethical Review Board of the Nagoya University School of Medicine (2021-0301). All participants provided written informed consent before participation.

### 2.2. Equipment

**Figure 1:**
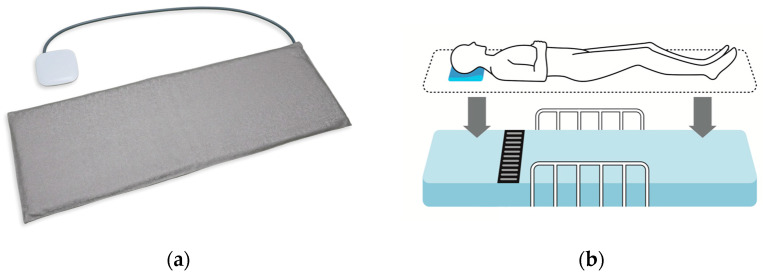
Mat-type sensor and placement methods. (**a**) The mat-type sensor (Techno Horizon Co., Ltd.) used in this study; (**b**) The mat-type sensor was placed under the mattress at chest level to measure pulse and respiratory rates during sleep.

A commercially available mat-type non-contact sensor, the Mimamori-fu CS-2000 (Techno Horizon Co., Ltd., Nagoya, Aichi, Japan), which has been certified for medical safety, was used in this study (Figure 1a). This device detects body and breathing movements using an air vibration sensor and calculates the respiratory rate and pulse rate at 1-min intervals. The air vibration detecting method used in this device does not need electronic components in the mat, making it more durable and less susceptible to external electrical noise than the other mat-type sensors (i.e., pressure or force sensors). Moreover, this method is advantageous in that it does not generate electromagnetic waves, making it safe to use for patients with pacemakers and other devices. The dimensions of the sensor mat used in this study were 835 mm in width, 19 mm in height, and 290 mm in depth. The sensor was placed under the mattress at chest level, and it was recorded continuously for three days (Figure 1b). To ensure standardized measurement conditions, only a common type of hospital bed mattress was adopted. We avoided pressure-dispersing mattresses to prevent pressure ulcers.

### 2.3. Respiratory Rate

Visual inspection by physical and occupational therapists was used as the gold standard for measuring respiratory rate. Once a day, the therapists measured the resting respiratory rate of each subject while they were lying in bed. For mat-type sensor measurements, the respiratory rate recorded at the same time as the visual inspection was extracted and used for analysis.

### 2.4. Pulse Rate

A cuff-type automated blood pressure device was used as the gold standard for measuring the pulse rate. Once a day, the physical and occupational therapists measured the pulse rate of each participant while lying in bed. For the mat-type sensor measurements, the pulse rate was recorded simultaneously as the automated blood pressure device measurement was extracted and used for analysis.

Additionally, for subjects who underwent 24-h Holter ECG (Cardiomemory RAC-3502, Nihon Kohden Corporation, Tokyo, Japan) as deemed necessary by the attending physician for medical purposes, the pulse rate obtained from the 24-h Holter ECG was also referenced. This device automatically calculates the average pulse rate per hour. Similarly, the average pulse rate for each hour was calculated from the mat-type sensor measurements and used for analysis. The data extraction period was from 19:00 to 7:00 the following morning and included night-time sleep.

### 2.5. Data Analysis

The respiratory and pulse rates obtained from the mat-type sensor were compared and validated against the respective gold-standard data. Data points where the mat-type sensor determined that the subject was out of bed were excluded from the analysis. First, the Bland–Altman analysis was used to quantify the differences between the mat-type sensor measurements and gold-standard measurements [25]. The Bland–Altman analysis is a statistical method that assesses the agreement between two different measurement techniques by plotting the differences between paired measurements against their mean values [26]. The mean difference and the Limits of Agreement (LOA: mean difference ± 1.96SD) were calculated. Additionally, the 95% confidence interval (95%CI) of the mean difference was calculated to determine its presence of mean difference. If the 95%CI did not include zero, the mean difference was considered statistically significant. To assess the presence of proportional bias, a regression equation was calculated for the Bland–Altman plot, and a significance test was performed. If the regression was significant, a proportional bias was considered to exist. The proportional bias suggests that the difference between the two methods varies depending on the magnitude of the measurements. A *t*-test was conducted to compare the mean differences between groups with and without arrhythmia. The significance level was set at *p* < 0.05. Pearson’s correlation coefficient was used to examine the correlation between the respiratory and pulse rates obtained from the mat-type sensor and the gold standard. The interpretation of the correlation coefficients was based on Guilford’s guidelines [27], with ±0.0 to ±0.2 indicating no correlation, ±0.2 to ±0.4 indicating a weak correlation, ±0.4 to ±0.7 indicating a moderate correlation, and ±0.7 to ±1 indicating a strong correlation. All statistical analyses were performed using the Python software (version 3.8).

## 3. Results

Forty-eight patients were included in this study. The mean age was 78.5 ± 10.0 years; 29 were male, and the mean weight was 59.0 ± 12.6 kg. Among the 48 subjects, 29 underwent 24-h Holter ECG. The mean age of this subgroup was 79 ± 10.8 years; 18 were male, and the mean weight was 59.4 ± 13.6 kg (Table 1). As the recommended weight for using this mat-type sensor ranges from approximately 40 kg to a maximum of 200 kg, the weight of these subjects fell within this range.

**Figure 2:**
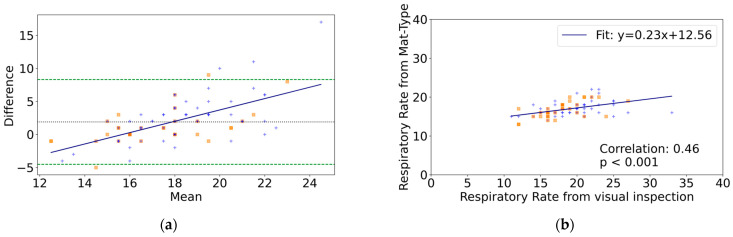
Comparison between the mat-type sensor and visual inspection (n = 48, number of datasets = 96). (**a**) Bland–Altman Plot of differences in respiratory rate measurements (visual inspection minus mat-type sensor). The dotted line indicates the mean difference. Dashed lines indicate the upper and lower limits of agreement (mean ± 1.96 standard deviations). The solid line represents the regression line. (**b**) Correlation plot of respiratory rate measurements between the mat-type sensor and visual inspection. Orange squares represent participants with arrhythmia, while blue crosses represent those without arrhythmia.

For the Bland–Altman analysis of respiratory rates obtained from the mat-type sensor compared to the visually obtained respiratory rates, 96 data points were analyzed. The mean difference was 1.9 respirations per minute (rpm) (95%CI, 1.2 to 2.6), and the LOA was −4.5 to 8.3 rpm, indicating the presence of a mean difference (Table 2). The regression was significant (r = 0.63, *p* < 0.05), indicating the presence of proportional bias (Table 2). In the group with arrhythmia, the mean difference was 1.1 rpm (95%CI, 0.19 to 2.0), and the LOA was −4.1 to 9.2 rpm. In the group without arrhythmia, the mean difference was 2.3 rpm (95%CI, 1.5 to 3.2), and the LOA was −4.5 to 9.2 rpm. There was no significant difference between the two groups (*p* = 0.05) (Figure 2a). The correlation coefficient between the respiratory rates obtained from the mat-type sensor and visually obtained respiratory rates was r = 0.46 (Figure 2b).

**Figure 3:**
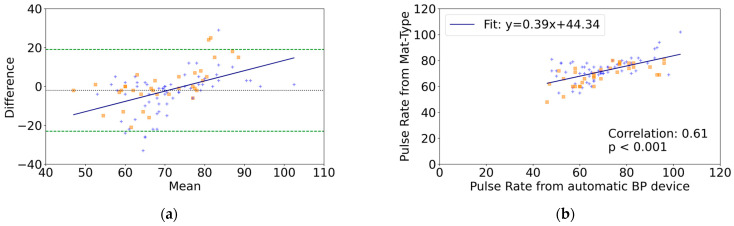
Pulse rate comparison between the mat-type sensor and the automated BP device (n = 48, number of datasets = 96). (**a**) Bland–Altman Plot of differences in pulse rate measurements (automatic BP device minus mat-type sensor). The dotted line indicates the mean difference. Dashed lines indicate the upper and lower limits of agreement (mean ± 1.96 standard deviations). The solid line represents the regression line. (**b**) Correlation plot of pulse rate measurements between the mat-type sensor and the automatic BP device. Orange squares represent participants with arrhythmia, while blue crosses represent those without arrhythmia.

For the Bland–Altman analysis of pulse rates obtained from the mat-type sensor compared to those obtained from the automated blood pressure device, 96 data points were analyzed. The mean difference was −2.0 beats per minute (bpm) (95%CI, −4.2 to 0.2), and the LOA was −23.0 to 19.0 bpm, indicating no significant mean difference (Table 2). The regression was significant (r = 0.49, *p* < 0.05), indicating the presence of proportional bias (Table 2). In the group with arrhythmia, the mean difference was 0.7 bpm (95%CI, −2.9 to 4.2), and the LOA was −19.7 to 21.0 bpm. In the group without arrhythmia, the mean difference was −3.5 bpm (95%CI, −6.2 to −0.8), and the LOA was −24.5 to 17.6 bpm. There was no statistically significant difference between the two groups (*p* = 0.07) (Figure 3a). The correlation coefficient between the pulse rates obtained from the mat-type sensor and those obtained from the automated blood pressure device was r = 0.61 (Figure 3b).

**Figure 4:**
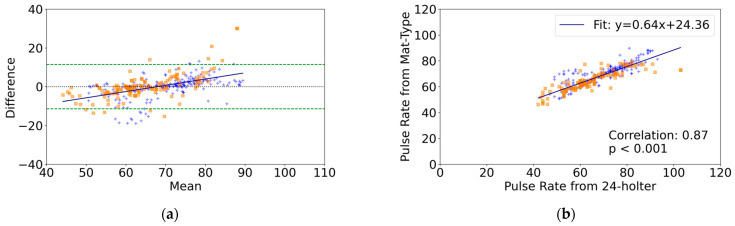
Pulse rate comparison between the mat-type sensor and 24 h Holter ECG (n = 29, number of datasets = 365). (**a**) Bland–Altman Plot of differences in pulse rate measurements (24 h Holter ECG minus mat-type Sensor). The dotted line indicates the mean difference. Dashed lines indicate the upper and lower limits of agreement (mean ± 1.96 standard deviations). The solid line represents the regression line. (**b**) Correlation plot of pulse rate measurements between the mat-type sensor and 24 h Holter ECG. Orange squares represent participants with arrhythmia, while blue crosses represent those without arrhythmia.

For the Bland-Altman analysis of pulse rates obtained from the mat-type sensor compared to those obtained from the 24-h Holter ECG, 365 data points were analyzed. The mean difference was 0.01 bpm (95%CI, −0.6 to 0.6), and the LOA was −11.4 to 11.4 bpm, indicating no significant mean difference (Table 2). The regression was significant (r = 0.52, *p* < 0.05), indicating the presence of proportional bias (Table 2). In the group with arrhythmia, the mean difference was 0.1 bpm (95%CI, −1.0 to 1.2), and the LOA was −12.4 to 12.6 bpm. In the group without arrhythmia, the mean difference was −0.04 bpm (95%CI, −0.8 to 0.7), and the LOA was −10.7 to 10.7 bpm. There was no significant difference between the two groups (*p* = 0.80) (Figure 4a). The correlation coefficient between the pulse rates obtained from the mat-type sensor and those obtained from the 24-h Holter ECG was r = 0.87 (Figure 4b).

## 4. Discussion

Our study results revealed that the mat-type noncontact sensor could achieve a certain level of accuracy compared with conventional clinical vital sign measurement methods among patients with acute-to-sub-acute-phase cardiovascular diseases. However, the mat-type sensor tended to overestimate respiratory rates above 20 rpm and pulse rates above 80 bpm. Conversely, the mat-type sensor tended to underestimate the pulse rates below 60 bpm. These findings suggest that despite a certain degree of error, the mat-type sensor can provide high-precision, long-term vital sign monitoring in the management of patients with cardiovascular diseases, thereby contributing to high-quality medical care.

Clinical assessments of respiratory rate have been reported to have a variability of ±6.2 rpm between observers [28], and our results demonstrated a level of accuracy similar to that of previous studies. Furthermore, the mat-type sensor maintained a consistent level of accuracy, regardless of the presence or absence of arrhythmia. These findings suggest that the mat-type sensor can measure the respiratory rate with an accuracy equivalent to that of clinical assessments and can sensitively detect changes in symptoms.

Detecting increases or decreases in the respiratory rate is crucial because it is the most sensitive indicator of severity and early clinical signs of acute illnesses [4,5]. However, respiratory rate is considered the most frequently missing data in medical records [7]. Without sufficient baseline records, it is challenging to identify the signs of symptom deterioration. This method can automatically measure and record respiratory rate with an accuracy equivalent to that of clinically used methods. Moreover, a significant advantage of the mat-type noncontact sensor is that it can be placed underneath a mattress, allowing measurements to be taken without patient awareness. This is also an essential factor for accurately measuring respiratory rates [29]. In contrast, other types of noncontact sensors, such as cameras and sensors, are installed in visible locations, which may influence the respiratory rate measurements. Therefore, the mat-type sensor used in this study can provide stable respiratory rate monitoring in clinical settings.

Pulse rate measurements in current clinical practice have been reported to have a variability of ±10.6 bpm between observers [28]. The results obtained from the automated blood pressure device used in this study showed a larger LOA than those reported in previous studies. However, the pulse rate obtained from the 24-h Holter ECG demonstrated accuracy levels similar to those reported in previous studies. Both results maintained a consistent level of accuracy regardless of the presence or absence of arrhythmia. The pulse rate can vary widely depending on an individual’s mental state at the time of measurement and their activity immediately before the measurement [30]. As this study included patients who could undergo rehabilitation interventions, it is possible that a certain level of physical activity was performed before pulse rate measurement using the automated blood pressure device, causing fluctuations in the pulse rate at the time of measurement, which was not adequately reflected by the mat-type sensor. In contrast, the pulse rate measured by the 24-h Holter ECG includes data from night-time sleep, which may exclude pulse rate fluctuations due to psychological and physical factors [11]. Moreover, calculating the average value every hour can minimize the impact of motion artifacts caused by body movements at night and the influence of activity. Therefore, the resting pulse rate during night-time sleep could be stably measured. Additionally, the ability to measure the resting pulse rate with high accuracy allows the estimation of the predicted maximum heart rate reserve, which can be used as a guide for exercise intensity. This can be a significant advantage for patients with cardiovascular diseases [10]. Furthermore, research has shown an association between resting pulse rate during night-time sleep and cardiovascular disease, mortality, and even the occurrence of cerebrovascular events. In particular, when the pulse rate increases, there are reports of a higher risk of future cardiovascular events and early mortality [9]. This information emphasizes the importance of night-time pulse rate monitoring in the management of patients with cardiovascular diseases. Consequently, night-time pulse rate monitoring using a mat-type sensor is expected to play a crucial role in the long-term health management and prognosis improvement of patients with cardiovascular diseases. However, it should be noted that the number of 29 patients evaluated using the 24 h Holter ECG does not reach the minimum sample size recommended in the preliminary power analysis (34 patients). This insufficient sample size may reduce the statistical power of the Holter ECG-based analysis and should be kept in mind when interpreting the results. However, the sample size was sufficient for the main analyses in this study, and the overall conclusions regarding the accuracy assessment of the mat-type non-contact sensor appear reasonable. Future studies should use larger sample sizes to further validate comparisons with Holter ECG.

The mat-type noncontact sensor tended to overestimate pulse and respiration rates when they were high and underestimate them when they were low. However, there was no tendency to incorrectly measure abnormal vital signs within the normal range, such as overestimation of low pulse rate or respiration rate or underestimation of high pulse or respiration rates. Therefore, the errors observed in this study were considered clinically acceptable. When using a mat-type non-contact sensor in clinical settings, if abnormal values are detected, it is necessary to keep these errors in mind and manually re-measure vital signs while carefully evaluating the patient’s condition. It should also be noted that our findings indicated LOAs of −4.5 to 8.3 for the respiratory rate and −23.0 to 19.0 for the pulse rate, which were larger than those reported in previous studies using similar mat-type sensors [21]. The differences in LOAs may be attributed to the controlled environmental conditions, such as temperature and humidity, and the exclusive use of data during sleep in the experiments. Although such a controlled environment may contribute to improved accuracy, it is difficult to replicate these optimal conditions in actual clinical practice. In contrast, our study demonstrated consistent accuracy for both respiratory rate and heart rate, regardless of the presence or absence of arrhythmia or the environment in which the sensor was used, highlighting its potential for real-world clinical applications.

Noncontact vital sign monitoring may reduce the opportunity for direct contact with patients, leading to the risk of missing information obtained through direct interaction with the patient [2]. Therefore, the use of noncontact sensors in patients with unstable symptoms should be carefully considered. However, when the patient’s overall condition is relatively stable, monitoring using non-contact sensors can minimize physical and psychological burdens on the patient during vital sign measurement, leading to improved compliance with vital sign monitoring. Additionally, it can reduce sleep disturbances associated with night-time vital sign measurements and the burden on medical staff. By monitoring resting vital signs at baseline, clinical signs and health status can be assessed, thereby promoting preventive medicine and early intervention. Noncontact vital sign monitoring technology is not intended to completely replace traditional measurement methods but rather to complement them, thereby providing higher-quality medical care. Furthermore, mat-type sensors can be used outside of hospitals. The simple installation method demonstrated in this study can be easily introduced in nursing homes or home environments with no technically proficient staff members. As this technology progresses, it will be possible to monitor vital signs remotely at a level similar to that of hospital assessments. In particular, daily vital sign monitoring at home is expected to improve the empowerment and self-management of individuals [31], and it is considered highly useful from the perspective of preventive medicine.

This study had several limitations. First, it is unclear how body movements affect vital sign measurements. It has been shown that the accuracy of noncontact sensors can be reduced by body movements and environmental factors [14]. In particular, body movements, body position and getting out of bed during night-time sleep can complicate the interpretation of pulse and respiratory rate measurement results. Future research should aim to improve measurement accuracy by specifically analyzing the impact of these factors. For example, using sensors with body movement detection capabilities to identify and exclude data when body movements occur or collecting data only under specific environmental conditions could achieve more accurate vital sign measurements. The second limitation is that this study used a mat-type non-contact sensor to measure the respiratory rate and compared the results with visually obtained assessments. However, this approach may yield different results compared to other advanced measurement techniques, such as PSG. Visual counting varies among observers; therefore, its reliability as a standard is not fully guaranteed [7]. To address this issue, studies comparing respiratory rate measurements obtained using mat-type noncontact sensors with other advanced measurement techniques, including PSG, are required. Third, it is important to note that mat-type sensors may not detect vibrations accurately in individuals with low body weight. Furthermore, the performance of these sensors may vary depending on the type of mattress and the patient’s medical conditions. Despite the promising results of this study, further research is needed to assess the generalizability of these findings to medically unstable patients or specialized bed conditions, such as pressure-dispersing mattresses. Despite these limitations, our current findings suggest that the noncontact mat-type sensor is suitable for use in clinical settings and can provide acceptable accuracy.

## 5. Conclusions

In this study, we verified that a mat-type noncontact sensor with an air vibration method could accurately calculate the respiratory rate and night-time sleeping pulse rate in patients with acute-to-sub-acute-phase cardiovascular diseases. This method can reduce the burden on patients and healthcare providers while allowing the monitoring of resting vital signs. These data are expected to contribute to the early identification of risks and the optimization of the timing of preventive interventions in patients with cardiovascular diseases. This study also contributes to future research aiming to improve the accuracy of mat-type sensors by combining other sensing techniques, such as body movement detection.

## Figures and Tables

**Table 1:** Clinical characteristics of the study participants.

	Participants for Referring HR *^1^ (n = 29)	All Participants (n = 48)
Clinical characteristics		
Age (years)	79 ± 10.8	78.5 ± 10.0
Gender (male/female)	18/11	29/19
Weight (kg)	59.4 ± 13.6	59.0 ± 12.6
Diagnosis		
Stroke		
Days from onset to start of evaluation	34.2 ± 8.1	32.7 ± 8.7
Cerebral infarction (%)	11 (37.9%)	16 (33.3%)
Hemorrhage (%)	2 (6.9%)	4 (8.3%)
Heart failure (%)	16 (55.2%)	28 (58.3%)
Days from admission to start of evaluation	12.9 ± 14.8	11.8 ± 12.4
Comorbidities		
Hypertension (%)	13 (44.8%)	23 (47.9%)
Diabetes mellitus (%)	6 (20.7%)	12 (25.0%)
Arrhythmia		
No arrhythmia	16 (55.2%)	25 (52.1%)
Frequent premature ventricular contraction (%)	4 (13.8%)	6 (12.5%)
Arterial fibrillation *^2^ (%)	5 (17.2%)	11 (22.9%)
Sinus arrhythmia (%)	3 (10.3%)	3 (6.25%)
Not specified (%)	1 (3.4%)	1 (2.1%)
Medication		
Beta-blockers (%)	10 (34.5%)	18 (37.5%)
Cilostazol (%)	1 (3.4%)	3 (6.3%)
MMSE-J, mean ± SD	26 ± 3.0	26 ± 3.0

*^1^ Participants who had a 24-h Holter ECG performed on the same day as the mat-type sensor; *^2^ Paroxysmal/persistent/permanent atrial fibrillation.

**Table 2:** Fixed and proportional bias in Bland–Altman analysis.

	Fixed Bias						Proportional Bias	
	Mean Difference	95%CI	Lower LOA	95%CI	Upper LOA	95%CI	Difference vs. Mean	
							r	*p*
Respiratory rate mat-type vs. visual inspection	1.9	1.2, 2.6	−4.5	−5.7, −3.4	8.3	7.2, 9.4	0.63	<0.05
Pulse rate mat-type vs. automated BP device	−2.0	−4.2, 0.2	−23.0	−26.8, −19.3	19.0	15.3, 22.8	0.49	<0.05
Pulse Rate mat-type vs. 24-h Holter ECG	0.01	−0.6, 0.6	−11.4	−12.4, −10.4	11.4	10.4, 12.5	0.52	<0.05

## Data Availability

The data presented in this study are available upon request from the corresponding author. The data are not publicly available due to privacy and ethical restrictions.
